# Wayfinding in Healthcare Facilities: Contributions from Environmental Psychology

**DOI:** 10.3390/bs4040423

**Published:** 2014-10-31

**Authors:** Ann Sloan Devlin

**Affiliations:** Department of Psychology, Connecticut College, 270 Mohegan Avenue, New London, CT 06320, USA; E-Mail: asdev@conncoll.edu; Tel.: +1-860-439-2333; Fax: +1-860-439-5300

**Keywords:** wayfinding, healthcare, technology, user characteristics

## Abstract

The ability to successfully navigate in healthcare facilities is an important goal for patients, visitors, and staff. Despite the fundamental nature of such behavior, it is not infrequent for planners to consider wayfinding only after the fact, once the building or building complex is complete. This review argues that more recognition is needed for the pivotal role of wayfinding in healthcare facilities. First, to provide context, the review presents a brief overview of the relationship between environmental psychology and healthcare facility design. Then, the core of the article covers advances in wayfinding research with an emphasis on healthcare environments, including the roles of plan configuration and manifest cues, technology, and user characteristics. Plan configuration and manifest cues, which appeared early on in wayfinding research, continue to play a role in wayfinding success and should inform design decisions. Such considerations are joined by emerging technologies (e.g., mobile applications, virtual reality, and computational models of wayfinding) as a way to both enhance our theoretical knowledge of wayfinding and advance its applications for users. Among the users discussed here are those with cognitive and/or visual challenges (e.g., Down syndrome, age-related decrements such as dementia, and limitations of vision). In addition, research on the role of cross-cultural comprehension and the effort to develop a system of universal healthcare symbols is included. The article concludes with a summary of the status of these advances and directions for future research.

## 1. Introduction: Environmental Psychology and Healthcare Facility Design

Research from environmental psychology has the chance to improve our lives; this claim is nowhere more evident than at the intersection of environmental psychology and healthcare facility design. In addition, this intersection reflects one of the primary characteristics of environmental psychology: its interdisciplinary quality. Much of the research on healthcare facility design involves collaborations between environmental researchers and those in other professions (e.g., architecture, computer science). Some of the contributions are applications to existing topics (e.g., wayfinding) in the context of healthcare; other findings are specific to healthcare (e.g., the effects of same-handed *vs.* mirror-image inpatient rooms). Reflecting the interdisciplinary nature of the discipline, literature about these issues is not only found in the mainstays of the discipline (*i.e.*, *Environment and Behavior* and the *Journal of Environmental Psychology*), but also in journals specifically developed for this subject matter (e.g., *Health Environments Research and Design Journal*). Beyond this inner core, research integrating environmental psychology and healthcare facility design is found in a wide array of journals indirectly related to the physical environment including nursing, critical care, pain, ergonomics, emergency medicine, comparative effectiveness research, intensive care, infectious diseases, and public health, among others. Research on wayfinding, the particular focus of this article, is also summarized in compendiums covering wayfinding in health care [1,2,3].

## 2. Wayfinding

Despite its fundamental role, wayfinding is often overlooked in the evidence-based design research, although Ulrich, Berry, Quan, and Parish [4] include it as one of their dimensions in a nine-faceted framework for evidence-based design. Reinforcing this assessment of wayfinding as an underappreciated aspect of the designed environment, a map designer quoted in Devlin’s [5] book about doctors’ offices points to the attitude of architects that wayfinding systems are often an afterthought and overlay. This map designer notes that wayfinding systems are infrequently part of the planning process at the programming stages. Facility planners are encouraged to use the master planning process to create effective wayfinding systems [6], but this advice is seldom heeded, despite the fact that wayfinding is one of the variables beyond clinical service that affect patients and staff [7,8]. This lack of recognition about the critical role of wayfinding systems has unfortunate outcomes because an environment that fosters independent wayfinding will reduce costs; people who are unsure where they are and how to reach a destination will interrupt staff engaged in other activities. For example, Nelson-Shulman [9] showed that patients exposed to posted signs in an admitting area made fewer demands on staff and were more knowledgeable about admitting procedures and amenities available, in contrast to patients without this posted information. A cost-estimate by Zimring [10], which is often cited, is that problems in wayfinding at Emory University Hospital cost the institution $220,000 annually. Most of the research using evidence-based design focuses on patients’ interactions with the clinical areas of the hospital, rather than on the more public spaces where wayfinding typically begins, despite the role of such spaces in the experience of patients and visitors.

An early application of wayfinding to the healthcare arena came from the work of Carpman, Grant, and Simmons [11], whose book *Design that Cares: Planning Health Facilities for Patients and Visitors* is a landmark volume that integrates environmental research and healthcare design. The authors argue that a “coordinated wayfinding system” is needed in healthcare facilities and that the ease of wayfinding will affect stress [11] (p. 19). Research from that book points to a number of themes that have received continued attention, including the importance of nomenclature (*i.e.*, how destinations are named), density (*i.e.*, the number of signs), context, placement, and visibility. Studies conducted for the University of Michigan Patient and Visitor Participation Project (PVP), part of the Replacement Hospital Program, generated much of the research for *Design that Cares* (both published papers and unpublished research reports) [12,13]. These documents look at such issues as the power of environmental affordance (what the environment “says” to us through its structure) *vs.* that of manifest cues (the effectiveness of the actual signage posted in the environment) [13].

A good deal of the research on wayfinding taps into the capacity of human cognition, including how much information we can hold in short term memory, for example the seminal article by Miller [14]; difficulties in multitasking [15]; and the schemas we have for the relationship between signage and the physical environment (e.g., that movement forward in space is up on a map) [16,17]. Research with applications for wayfinding in healthcare environments has often come from other institutional settings such as housing for the elderly or long-term care facilities [18,19] and libraries [20], although beyond Carpman *et al.* [11,12,13] there is some early work on healthcare environments [21,22,23]. From the standpoint of plan configuration and signage, wayfinding research on any large building or complex of buildings is applicable to healthcare environments.

### 2.1. Plan Configuration and Manifest Cues 

Wayfinding is a particular challenge in large healthcare complexes with numerous buildings [7], often lacking distinctive appearance, which are linked to one another as the complex grows over time. Early on in the research on wayfinding, plan configuration was shown to be a correlate of wayfinding performance [24,25]. More recent research substantiates that finding. Using architecture students as newcomers to polyclinics that differed in their symmetry, Baskaya, Wilson, and Özcan [26] used reactions to a tour and a sketch map task to show the benefits of a regular but asymmetrical setting over a regular and symmetrical setting. Strikingly, 63.2% of the participants in the regular, symmetrical building felt “completely lost” during a tour in contrast to only 6.5% of those in the regular, asymmetrical setting [26] (p. 851). Baskaya *et al.* [26] showed that symmetry and repetition of similar elements could be a drawback to wayfinding, pointing again to layout as an element to be considered in the initial plan. The authors remark that landmarks to create distinctiveness may be particularly important in a building with symmetry. At the neural level, researchers are developing more sophisticated explanations of how landmarks may be coded, and the spatial layout itself has been described as a kind of landmark [27].

As the comment by Baskaya *et al.* [26] shows, wayfinding includes both attention to the floor plan (the building structure) and environmental cues (e.g., landmarks, signage) overlaid on that floor plan [28]. These distinctions are also reminiscent of the idea introduced by Carpman *et al.* [13] that we have the environmental affordance (what the structure suggests can occur) and the manifest cues (e.g., signage) that are used to elucidate the floor plan configuration. Some research [29] has placed environmental affordances (*i.e.*, corridor width, brightness) in direction competition with manifest cues (*i.e.*, signage), showing that both of these aspects play a role under varying circumstances (*i.e.*, for everyday location tasks *vs.* emergencies).

These differences between the role of the floor plan and of manifest cues were illustrated in research on individuals with dementia [28], showing which features support or interfere with their wayfinding. Marquardt mentions long corridors, repetitive elements, and changes of direction within the circulation system as structural aspects that negatively affect orientation; information clutter is mentioned as an environmental design feature that does so as well. Considering the philosophy of universal design, which advocates that all environments should be designed “with the widest range of users in mind” [30] (paragraph 1), this research from individuals with dementia should be applied to users at every level of functioning. Related to structure, there is some suggestion that limiting choice (e.g., fewer choices at nodes; fewer rather than more routes and corridors) may promote successful wayfinding [31,32]. The research by Cubukcu and Nasar and Slone *et al.* introduces an approach that is increasingly used to test wayfinding behaviors for environments: virtual reality (VR). Although the virtual environments used in the Slone *et al.* [32] research were based on an academic building, they in many ways resemble an institutional setting such as a hospital.

Another aspect of plan configuration that merits consideration is the distinction between horizontality and verticality [33]. Floors that are stacked, which is commonly the case in multi-level public buildings, present particular challenges for wayfinding. The difficulty arises because these floors are typically perceived only indirectly, unless such design features as a central atrium make a visual reference between floors possible [33]. Understanding such challenges could inform design decisions that facilitate successful wayfinding.

### 2.2. Technological Approaches: Virtual Reality (VR), Head-mounted Displays (HMD), Mobile Technologies, and Touch Screen Monitors

More recent advances in applications of research on wayfinding to healthcare design often involve virtual reality (VR). VR provides a degree of experimental control that is frequently missing in field studies and allows researchers to isolate particular variables before these are tested in the field. A number of authors have used virtual environments for research on wayfinding with implications for healthcare [34,35,36,37,38,39].

Some of this research has direct implications for health and safety. For example, Tang, Wu and Lin [38] used virtual reality to examine emergency egress and demonstrated a significant improvement overall in emergency egress speed when signs were posted [either the old version (graphics and use of the word “emergency direction”) or the new version (graphics and use of the word “exit”)] in contrast to wayfinding without signs. Relative to the idea that people operate on the basis of schemas (which may reflect environmental affordances), the research also showed that over 40% of the participants chose an exit door rather than following the direction posted on the emergency sign when the choice was available.

VR provides the opportunity to examine specific independent variables in a controlled manner. For example, a VR video simulated a healthcare facility with a base model and a more elaborate model, which differed in the number of architectural aids available. These models were tested with younger adults (college-aged students) and older adults (ages 66–82) on a simulated tour (video 1) from the radiology waiting area to the radiology department [40]. Following exposure to each of three return videos (videos 2, 3 and 4), participants were asked which direction they would select at a designated choice point (where the video was stopped) to return to the origin. Younger adults were in general more successful than were older adults at selecting the correct direction to follow; older adults seemed to rely more on wayfinding cues with impact or salience, such as large colorful logos, than did younger adults. More limited memory function in the elderly points to the need to provide more frequent salient landmarks for wayfinding, according to the authors.

In addition to signage, VR has been used to study interconnection density (ICD), a concept related to plan configuration. ICD is “the average number of connected decision points for each decision point in a particular environment” [39] (p. 463). Generally, higher ICD is correlated with more problems in wayfinding, but the authors point out that perceived figural complexity can be dissociated from wayfinding difficulty [39] (p. 464). That is, according to the authors, two environments might have the same ICD but vary in their floor plans. For example, a linear hallway and a spiral hallway—think of the Guggenheim—both have the same ICD. Werner and Schindler varied two dimensions (1) alignment between the region near an elevator and the rest of the building and (2) type of corner, clipped (corners at less than 90 degrees) *vs.* orthogonal (corners at 90 degrees). In a between subjects design, one of four different virtual environments created from these dimensions (alignment and corner type) of an office building was displayed on a computer monitor. Participants (college students) were randomly assigned to one floor plan from this 2 × 2 design and completed 30 different wayfinding tasks to find target locations. Dependent variables included time elapsed on these wayfinding tasks; a pointing task; and selection of a floor plan that reflected their experimental condition. Results indicated significantly slower performance when the region around the elevator “was misaligned with respect to the rest of the building” [39] (p. 477). There were also large pointing errors; and participants had trouble selecting the floor plan that represented their experimental condition. The authors suggest ways to reduce the cognitive load that misaligned configurations create, including the use of a vista and outside landscaping to help provide a global frame of reference.

Related to ICD is the concept of space syntax, an approach that analyzes spatial configurations (*i.e.*, layouts) by measuring their connectivity and integration. Space syntax has been used to describe healthcare settings, such as predicting the movement of nurses in hospital units [41]. Haq and Luo [42] provide a comprehensive review of this work, noting that the largest number of articles reporting use of this technology in healthcare settings involved wayfinding [42] (p. 99). Haq and Luo point out that space syntax is typically used in healthcare settings to “quantify the environment as a set of predictor variables for a specific behavior” [42] (p. 100). Among the wayfinding topics addressed using space syntax has been visitor behavior in public areas, which showed that when in doubt (*i.e.*, uncertain about location) people gravitate to spaces with higher integration (*i.e.*, spaces that are more connected to other spaces) [43]. This principle has been demonstrated in a variety of building layouts [44] and points to the importance of spaces with integration (*i.e.*, that are highly connected to other spaces) to support wayfinding.

Another technology that is being used to learn more about wayfinding behavior involves head-mounted displays (HMD). Wilson and Wright [45] showed that first responders using these HMDs (integrated into firefighters’ masks) formed better cognitive maps than did those without the HMDs. On a range of dependent variables including course completion time, shorter distance traveled, and fewer navigation errors, participants (8 firefighters and 13 others) demonstrated superior performance with the HMDs, which showed their real-time location on a floor plan of university buildings, where the research took place. Not only does the ease of wayfinding have implications for patients and visitors, but first responders are also faced with challenges in the intricate complex of buildings often found on healthcare campuses.

Mobile technology is also being used to assist in wayfinding, demonstrated in a case study of Boston Children’s Hospital [46]. MyWay is a mobile application produced by Meridian to access hospital maps and locate the user within the facility through GPS via smartphones, with turn-by-turn steps. Wayfinding is very challenging in the Boston Children’s Hospital complex, which has 12 separate buildings (some interconnected, some detached), constructed over a 150-year span, with 6 campuses. The MyWay application provides more than locations, also showing photographs of the clinicians and their specialties. Meridian reports more than 4500 downloads in 6 months after the application’s launch; reports of 65% of users said it improved their experience in the hospital. Just under half of visitors to the hospital report having smart phones. This technology awaits evaluation via randomized controlled trials, but it provides one approach for wayfinding assistance in complex healthcare environments. Such indoor positioning technologies are becoming more available (see for example, the Wifarer™ system) [47].

As a pilot and research project, Wright *et al.* [48] used a touch screen monitor in the 3rd largest hospital in the UK to enable users to find 16 destinations that had been pre-selected primarily based on frequency of use. Results of 22 users whose movements were observed indicated a high rate of success (86%) in finding the selected destination. Given the rapid rate of change in technology and the rate of adoption of technology by users, it is likely that institutions such as hospitals will gravitate toward smart phone apps such as Meridian’s or the Wifarer™ system rather than investing in kiosks to provide support for wayfinding. Lending support to that prediction, data from the Pew Research Internet Project [49] indicate that as of January 2014, 90% of American adults had a cell phone, and 58% of American adults had a smart phone. The report indicates that of those with a cell phone, 49% use their device to “get directions, recommendations, or other location-based information”. 

In looking at the literature, there is a considerable emphasis on devices using auditory and/or haptic feedback that serve those with cognitive and/or sensory impairments [50,51,52,53] and less emphasis on those without such challenges. Legge *et al.* [52] present a Digital Sign System (DSS) providing location sensing by using digital signs (tags) that are detected by a reader held by the user. The major difference between the sighted controls and those with visual impairments was the speed of locating targets. This research focusing on those with challenges has obvious benefits for a wide array of users. For example, in an environment with a proliferation of visual signage, a system offering auditory and/or haptic feedback might facilitate wayfinding for all users.

Significant technologies are being developed to enable blind persons to navigate more independently in unfamiliar indoor environments. One such system uses object detection and text recognition, employing optical character recognition software available in the marketplace, which is then translated into speech for the user [54]. This technology has successfully used an algorithm for geometric shape to detect the location of such architectural elements as doors and elevators. 

In other advances, a recent wayfinding application for blind users “Blind-App Launcher” uses a smartphone platform to guide “visually impaired people from one place to [an]other providing directional, compass-like information in a universal way” [55] (p. 7214). The system provides visual (text, map), auditory, and tactile information; participants preferred a combination of auditory and tactile feedback.

Other recent approaches include what is known as a personalized accessibility map (PAM), which has been developed to aid those with disabilities to independently navigate in the environment [56]. This system developed at the University of Pittsburgh (PAM-Pitt) provides an advance over existing approaches mentioned in the article (e.g., Access Together, AXSMap, Planat, Rollsquare, and Wheelmap) offered in other geo-crowdsourcing applications. Geo-crowdsourcing involves the contributions of online users to document the accessibility of a range of spaces, such as public buildings and neighborhoods. The new system supports those with a range of challenges, including those of mobility, vision, and audition. PAM offers an advance over existing systems because it is “oriented towards the creation of a base map of accessibility features and providing personalized routing solutions based on a sidewalk network” [56] (p. 101).

More theoretically-oriented research also contributes to our understanding of the challenges in wayfinding. For example, the development of an allocentric cognitive map-based model of human wayfinding [57] has highlighted particular aspects of environments that are likely to be difficult to incorporate in cognitive maps. Called Magellan, after the Portuguese explorer, the model was tested with spatial navigation tasks (called *Yellow Cab*) set in virtual towns that included multistory office buildings and one-story retail establishments. The tasks involved finding efficient paths from a pick up point to a destination for passengers who had been randomly placed within the virtual town. Difficulty of finding paths increases with inter-store distance, even accounting for the spread of stores across the town. The researchers suggest that in real world wayfinding, more attention is likely paid to the role of landmark distinctiveness, a variable that future work on the model could address. The authors also offer the possibility that this model could be used to evaluate the wayfinding behavior of populations that might vary in age and cognitive challenges, which would offer insight into the rates of learning in such spatial navigation tasks while providing a standard against which optimum performance can be measured. 

### 2.3. Participant Characteristics

As the research on those with visual impairments by Legge *et al.* [52] and others suggests, a focus of research on wayfinding has addressed the needs of those with cognitive challenges such as dementia or sensory challenges such as visual impairment. There has been a particular emphasis on developing wayfinding systems that accommodate those with visual impairments, given that sight is our primary sensory modality. Frequently research has used technology that simulates visual impairment to provide experimental control and better understand the consequences for wayfinding. For example, using vision simulator goggles with normally sighted people to simulate the experience of five different kinds of visual impairment (e.g., cataracts), Rousek, Koneczny, and Hallbeck [58] pointed to difficulties in wayfinding related to (1) decorative elements (e.g., shiny floor tiles), (2) lighting that may be misleading (too bright or dim) (3) signage size (too small) and placement (in unexpected positions) as well as a number of other factors. Even without the goggles a substantial percentage of the participants reported wayfinding difficulties. A number of the comments from participants pointed to the expectations that people have about how signage is used in the environment (its location, size, and illumination). These results suggest designers could better understand people’s expectations (schemas) about environmental design and capitalize on this knowledge in wayfinding systems. For example, over 25% of the participants were confused that corners were not always at the expected 90 degrees. Hallways inherently afford guidance because they are directional—whereas large open areas present particular challenges for wayfinding. Even beyond adherence to the guidelines from the Americans with Disabilities Act (ADA) and the ADAAG (ADA Accessibility Guidelines) that the authors note, designers might give greater consideration to our expectations about the properties of environments, both through our schemas and through the affordances environments communicate.

In addition to examining the effects of visual limitations on wayfinding, investigators have used participants with Down syndrome in research on wayfinding (e.g., route learning), given the hypothesis of some that visuo-spatial ability may be a strength of those with Down syndrome. Pursuing this line of reasoning, a review of four studies on wayfinding with individuals who have Down syndrome in fact suggests limitations in wayfinding abilities, although the authors express concern about factors related to the internal validity of the small number of studies and call for more research [59]. Other research points to a reliance on route rather than configurational knowledge in Down syndrome individuals [60]. Research using VR with Down syndrome individuals demonstrated their ability to learn routes within a virtual town (3 buildings and 17 landmarks located in a grid of streets), but some limitation in locating a shortcut between two locations that were known [60]. Further, more trials were required for Down syndrome individuals to learn routes than was true of their chronological controls. Such findings have applications for learning in complex environments, where more time (practice) may be needed for Down syndrome individuals to master routes and the importance of reliance on such routes rather than expecting the development of configurational knowledge.

Beyond specific challenges such as Down syndrome and visual impairment, changes in cognitive abilities during normal aging may affect a number of aspects of wayfinding, including preferences for the colors used as the background on signage [61]. In addition, it appears that older individuals have considerable difficulty switching from an egocentric orientation of the environment (*i.e.*, viewer centered related to the body’s changing position) to an allocentric orientation (*i.e.*, based on an external coordinate system). Such difficulty among the elderly has implications for wayfinding success, in terms of demonstrating flexibility (e.g., short cuts) in route selection [62]. From the standpoint of safety, such difficulty may have implications for selecting routes in emergency situations.

Beyond cognitive and sensory challenges, other user issues relate to cross-cultural understanding, and in particular the search for symbols that can be understood across cultures. With the increasing diversification of the population in the US and other countries, particular attention is needed to communicate meaning through symbols, although opinions differ about the minimum level of universal comprehensibility a symbol should have [63]. While text can improve the comprehension of signage [64], text is also language-specific and thus limiting. 

One approach to increase cross-cultural comprehension in healthcare settings is to use universal healthcare symbols [65]. Lee *et al.* [65] showed that some symbols are well understood cross-culturally (for billing, ob clinic, and radiology), whereas others are not (for pharmacy, immunization, and family medicine). Their research included participants from the US, South Korea, and Turkey; countries were chosen to reflect “three distinct cultures” [65] (p. 879). Moreover, the symbols were taken from the set developed in the Hablamos Juntos (“we speak together”) project, funded by the Robert Wood Johnson Foundation, to develop a set of universal healthcare symbols. Even when symbols have been developed through previous research, as was the case here, there was far less than complete comprehension of their meaning.

In their research on understanding healthcare pictograms and visual impairment (created in normally-sighted people through vision simulators), Rousek and Hallbeck [66] cite data from the Robert Wood Johnson Foundation noting how much new hospital construction and renovation there will be (an estimate of $200 billion in the decade preceding 2014) and with that construction the associated need for effective wayfinding systems. The research of Rousek and Hallbeck clearly shows the problems people have interpreting abstract signage, particularly when unique features of a specific department are absent because the signage has been oversimplified. Their research further showed that the use of human figures could be effective in healthcare signage; the figures that were comprehended at a high level had something in common: “… the human body or body parts performing a specific action with a distinguishable feature, when needed (e.g., including a scalpel in a surgery pictogram)” [66] (p. 781).

Hashim, Alkaabi and Bharwani [67] also point to the need to test signage with participants from a range of cultures as well as age and literacy ranges. Their research employed the symbols developed in the Hablamos Juntos project. Out of 100 participants in their research, 80 were of Arabic cultural background, 6 were African, 6 were South Asian, and 8 were European/North American and Other, with 53 indicating they needed help filling out medical forms. Sixty were 25 years or younger, and only 9 were 46 or older; 84 were women. People had more difficulty interpreting specific healthcare symbols (e.g., Oncology) than general symbols (e.g., Coffee shop). The highest symbol recognition (66/100) was for pediatrics; the lowest (2/100) was for Oncology, despite the careful evaluation used to develop these symbols. The results suggest the need to test the symbols on participants varying along a wide spectrum of education; those with higher levels of education were likely to have high recognition. “In an era of global health, massive international travel and highly multicultural communities, there remains a pressing need for easily recognizable, universal way-finding signs for healthcare facilities” [67] (p. 509). Problems occur with the use of monolingual text, religious references (like the cross), and trademarks, such as the Red Cross. In this symbol set, the teddy bear with a cross situated in its midsection was used as a symbol for pediatrics, but the authors comment this representation is unlikely to be meaningful outside of North America.

## 3. Summary and Conclusions

The research reviewed here focused on (1) the role of plan configuration *vs.* manifest cues in wayfinding (2) the emerging technologies used both to study wayfinding (e.g., virtual reality), on the one hand, and to provide wayfinding information (e.g., mobile apps), on the other (3) the challenges in wayfinding related to cognitive and visual impairments and (4) the search for universal healthcare symbols as countries become increasingly diversified. Table 1 presents a summary of the research findings for Section 2.1, Section 2.2 and Section 2.3 (see Table 1).

With regard to the plan configuration and manifest cues, the research suggests there is a tension between people’s understanding of regular environments (that is, not misaligned) on the one hand, and the importance of creating distinctiveness in such predictable environments, on the other. The use of virtual reality is likely to grow in importance as a research tool, and mobile applications will become increasingly viable as wayfinding aids, which points to the importance of controlled research using this technology. As the population continues to age, research on both cognitive and visual impairments, among other challenges of aging, must include how wayfinding in healthcare environments is affected. Finally, the search for universal healthcare symbols needs further study, as current research shows considerable misunderstanding of the symbols developed from the Hablamos Juntos (“we speak together”) project.

**Table 1:** Summary of Research Findings for Section 2.1, Section 2.2 and Section 2.3.

Section	References	Summary
2.1	[13,24,25,26,28,29,31,32,33]	Plan configuration has an impact on wayfinding; judged simplicity is correlated to ease of wayfinding. At the same time, symmetry and repetition may lead to confusion, and distinctive landmarks emerge as an aid to lessen disorientation. Both environmental affordances (what the layout affords or makes possible) and manifest cues (signage) support wayfinding. Building verticality (stacked floors) also present challenges to wayfinding and architectural features (e.g., a glass atrium) may provide needed perceptual access. Individuals with cognitive challenges have particular difficulty with repetitive elements and information clutter. Wayfinding for the elderly may be enhanced with more salient landmarks.
2.2	[34,35,36,37,38,39,40,41,42,43,44,45,46,47,48,50,51,52,53,54,55,56,57]	A range of new technologies provides advances in research on wayfinding, joining experimental control with ecological validity. These technologies include virtual reality, space syntax, head-mounted displays, location sensing devices, mobile devices for blind users that provide directional information, use of optical character recognition that can be translated into speech, and personalized accessibility maps that support individuals with challenges of mobility, vision, and audition. These technologies can improve health and safety (e.g., through more legible emergency egress; the use of vistas to offer a frame of reference; the importance of highly connected spaces) and provide technical support for independent wayfinding. Mobile technologies may support patient satisfaction through improved wayfinding experiences in complex hospital settings. Research points to differences in memory capacity for older *vs.* younger users, which in turn points to the need for universal design considerations for every user.
2.3	[58,59,60,61,62,63,64,65,66,67]	Wayfinding research has focused on those with challenges including cognitive and sensory limitations, with a particular emphasis on those with limitations of vision. Lessons learned from such research (e.g., drawbacks to lighting that is too bright or dim; highly reflective decorative elements; size and placement of signage) again point to the need for universal design considerations. VR research with Down syndrome individuals points to a reliance on routes and the absence of configurational knowledge. Similarly, older individuals have difficulty switching from egocentric to allocentric perspectives; this lack of flexibility points to safety issues in emergency situations when main routes may be blocked. Given the increasing need to communicate with a wide range of cultures that use healthcare facilities, research has developed a set of universal healthcare symbols. This project (Hablamos Juntos or “we speak together”) has met with some success, but there are limitations in the perception of abstract symbols. Those symbols based on the human form performing a specific action (e.g., a surgery pictogram with a figure, scalpel in hand) have been more widely understood.
